# A parameter-free learning automaton scheme

**DOI:** 10.3389/fnbot.2022.999658

**Published:** 2022-09-23

**Authors:** Xudie Ren, Shenghong Li, Hao Ge

**Affiliations:** ^1^School of Electronic Information and Electrical Engineering, Shanghai Jiao Tong University, Shanghai, China; ^2^Shanghai Data Miracle Intelligent Technology Co., Ltd., Shanghai, China

**Keywords:** parameter-free, Monte-Carlo simulation, Bayesian inference, learning automaton, parameter tuning

## Abstract

For a learning automaton, a proper configuration of the learning parameters is crucial. To ensure stable and reliable performance in stochastic environments, manual parameter tuning is necessary for existing LA schemes, but the tuning procedure is time-consuming and interaction-costing. It is a fatal limitation for LA-based applications, especially for those environments where the interactions are expensive. In this paper, we propose a parameter-free learning automaton (PFLA) scheme to avoid parameter tuning by a Bayesian inference method. In contrast to existing schemes where the parameters must be carefully tuned according to the environment, PFLA works well with a set of consistent parameters in various environments. This intriguing property dramatically reduces the difficulty of applying a learning automaton to an unknown stochastic environment. A rigorous proof of ϵ-optimality for the proposed scheme is provided and numeric experiments are carried out on benchmark environments to verify its effectiveness. The results show that, without any parameter tuning cost, the proposed PFLA can achieve a competitive performance compared with other well-tuned schemes and outperform untuned schemes on the consistency of performance.

## 1. Introduction

Learning Automata (LA) are simple self-adaptive decision units that were firstly investigated to mimic the learning behavior of natural organisms (Narendra and Thathachar, 1974). The pioneering work can be traced back to the 1960s by the Soviet scholar (Tsetlin, 1961, 1973). Since then, LA has been extensively explored and it is still under investigation as well in methodological aspects (Agache and Oommen, 2002; Papadimitriou et al., 2004; Zhang et al., 2013, 2014; Ge et al., 2015a; Jiang et al., 2015) as in concrete applications (Song et al., 2007; Horn and Oommen, 2010; Oommen and Hashem, 2010; Cuevas et al., 2013; Yazidi et al., 2013; Misra et al., 2014; Kumar et al., 2015; Vahidipour et al., 2015). One intriguing property that popularizes the learning automata-based approaches in engineering is that LA can learn the stochastic characteristics of the external environment it interacts with, and maximize the long-term reward it obtains through interacting with the environment. For a detailed overview of LA, one may refer to a new comprehensive survey (Oommen and Misra, 2009) and a classic book (Narendra and Thathachar, 2012).

**Figure 1:**
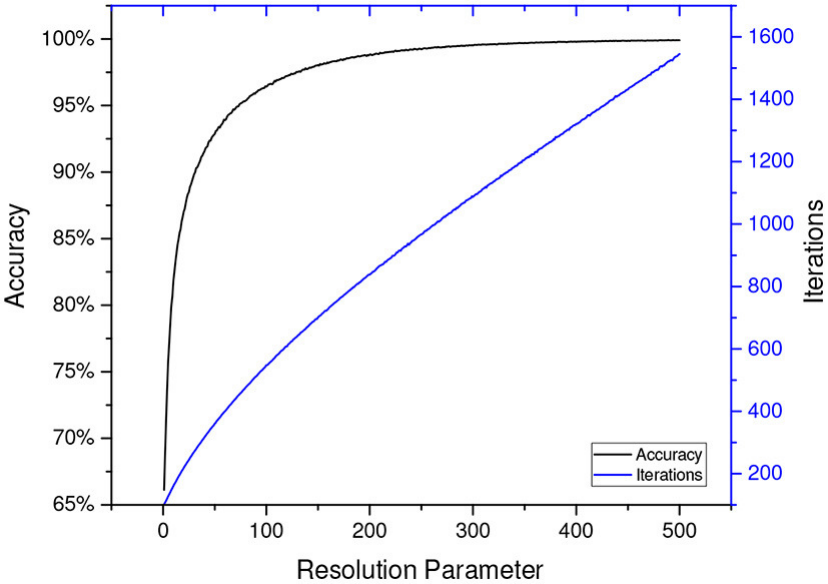
The accuracy and iterations with different resolution parameters for DP_*ri*_ (Oommen and Lanctôt, 1990) in benchmark environment *E*_1_, which is defined in Papadimitriou et al. (2004). The results are averaged over 250,000 replications.^2^

In the case of LA, *accuracy* and *convergence rate* become two major measurements to evaluate the effectiveness of a LA scheme. The former is defined as the probability of a correct convergence and the latter as the average iterations for a LA to get converged^1^. Most of the reported schemes in the field of LA have two or more tunable parameters, making themselves capable of adapting to a particular environment. An automaton's accuracy and convergence rate highly depend on the selection of those parameters. Generally, ensuring a high accuracy is of uppermost priority. According to the ϵ*-optimality* property of LA, the probability of converging to the optimal action can be arbitrarily close to one, as long as the learning resolution is large enough. However, it will raise another problem. Taking the classic Pursuit scheme for example, as Figure 1 illustrates, the number of iterations required for convergence grows nearly linearly with the resolution parameter, while the accuracy grows logarithmically. This implies a larger learning resolution can lead to higher accuracy, but at the cost of much more interactions with the environment. This dilemma necessitates parameter tuning to find a balance between convergence rate and accuracy.

In literature, the performance of various LA schemes is evaluated by comparing their convergence rates on the premise of a certain accuracy. The learning parameters of various schemes are tuned through a standard procedure to ensure the accuracies are kept at the same level, so that the convergence rates can be fairly compared. For deterministic estimator-based learning automata, the smallest value of the resolution parameter that yielded a hundred percent accuracy in a certain number of experiments is selected. The situation is more sophisticated when concerning the stochastic estimator-based schemes (Papadimitriou et al., 2004; Ge et al., 2015a; Jiang et al., 2015), because extra configurable parameters should be set to control the perturbation added. Parameter tuning is intended to balance the trade-off between speed and accuracy. However, the interaction cost can be tremendous itself^3^, due to its trial and error nature. In practical applications, especially where interacting with environments could be expensive, e.g., drug trials, destructive tests, and financial investments, the enormous cost for parameter tuning is undesired. Therefore, we believe, the issue of learning parameter configurations deserves more attention in the community, which gives impetus to our work.

The scope of this research is confined to designing a learning scheme for LA in which the parameter tuning can be omitted, and that's why it is called *parameter-free* in the title. It is noted that the term *parameter-free* does not imply that no configurable parameters are involved in the proposed model, but indicates a set of parameters for the scheme that can be universally applicable to all environments. This paper is an extension of our preliminary work (Ge et al., 2015b). The proposed scheme in Ge et al. (2015b) can only operate in two-action environments, whereas in this paper, our proposed scheme can operate in both two-action environments as well as multi-action environments. In addition, in this paper, optimistic initial values are utilized to improve the performance further. Moreover, a rigorous theoretical analysis of the proposed scheme and a comprehensive comparison among recently proposed LA schemes are provided in this paper which was not included in Ge et al. (2015b).

The contribution of this paper can be summarized as follows:

1. To the best of our knowledge, we present the first *parameter-free* scheme in the field of LA, for learning in any stationary P-model stochastic environment. The meaning of the terminology *parameter-free* is two-fold: (1) The learning parameters do not need to be manually configured. (2) Unlike other estimator-based schemes, initializations of estimators are also unnecessary in our scheme.
2. Most conventional LA schemes in literature employ a stochastic exploration strategy, on the contrary, we design a deterministic gradient descent-like method instead of probability matching as the exploration strategy to further accelerate the convergence rate of the automaton.
3. The statistics behavior of the proposed parameter-free learning automata (PFLA) is analyzed and rigorous proof of the ϵ-optimality property is provided as well.
4. Comprehensive comparison among recently proposed LA schemes is given to validate the theoretical analyses and demonstrate that PFLA is superior to other methods concerning tuning cost.

This paper proceeds as follows. Section 2 describes our philosophy and some related works. Section 3 presents the primary results of the paper: a parameter-free learning automaton scheme. Section 4 discusses the theoretical performance of the proposed scheme. Section 5 provides a numerical simulation for verifying the proposed scheme. Finally, Section 6 concludes this paper.

## 2. Related works

Consider a P-model environment which could be mathematically defined by a triple < 𝔸, 𝔹, ℂ >, where

- 𝔸 = {*a*_1_, *a*_2_, …, *a*_*r*_} represents a finite action set
- 𝔹 = {0, 1} denotes a binary response set
- ℂ = {*c*_1_, *c*_2_, …, *c*_*r*_} is a set of reward probabilities corresponding to 𝔸, which means Pr{*a*_*i*_ gets rewarded}=*c*_*i*_. Each *c*_*i*_ is assumed to lie in the open interval (0, 1).

Some other major notations that are used throughout this paper are defined in Table 1.

**Table 1:** Notations used in this paper.

**Symbol**	**Explanation**
*r*	The cardinality of the action set 𝔸
*E*	A vector of estimates
*N*	The number of repetitions in the Monte Carlo simulation
η	The threshold to terminate the iteration
*a*_*i*_	The *i*th action in 𝔸
α_*i*_	A parameter of *a*_*i*_'s beta distribution
β_*i*_	A parameter of *a*_*i*_'s beta distribution
*S*_*i*_	The number of times that *a*_*i*_ has been selected
$H_{i}$	The hypothesis that *a*_*i*_ is the optimal action
*Beta*(α, β)	A beta distribution with parameter α and β
*Norm*(μ, σ)	A normal distribution with mean μ and variance σ^2^
*B*(α, β)	The beta function
*B*(*x*; α, β)	The incomplete beta function

The aim of LA is to identify the optimal action *a*_*m*_, which has the maximum reward probability, from 𝔸 through interacting with the environment. The general philosophy is to collect feedback from the environment and use this information to extract evidence that supports an optimal assertion.

Then we are faced with two challenges:

1. How to organize the information gathered and make full use of them?
2. When is the time to make an assertion that claims one of the actions is optimal?

### 2.1. Information utilization

Lots of work have been done for the first challenge. Although the reward probabilities ℂ are unknown to us, we can construct consistent estimators to guarantee that the estimates of the reward probabilities can converge to their true values as the quantity of samples increases.

As the feedback for one action can be modeled as a Bernoulli distributed random variable in P-model environments, there are two ways to construct such estimators currently.

1. One is from the frequentist's perspective. The most intuitive approach is to utilize the likelihood function, which is a basic quantitative measure over a set of predictions with respect to observed data. In the context of parameter estimation, the likelihood function is naturally viewed as a function of the parameters *c*_*i*_ to be estimated. The parameter that maximizes the likelihood of the observed data is referred to as the *maximum likelihood estimate* (MLE). MLE-based LA (Oommen and Lanctôt, 1990; Agache and Oommen, 2002) are proved to be a great success, achieving a tremendous improvement in the rate of convergence compared with traditional variable structure stochastic automata. However, as we revealed in Ge et al. (2015a), MLE suffers from one principle weakness, i.e., MLE is unreliable when the quantity of samples is small.
  Several efforts have been devoted to improving MLE. The concept of *stochastic estimator* was employed in Papadimitriou et al. (2004) so that the influence of lacking samples can be reduced by introducing controlled randomnesses to MLE. In Ge et al. (2015a), we proposed an interval estimator-based learning automata DGCPA, in which the upper bound of a 99% confidence interval of *c*_*i*_ is used as estimates of reward probabilities. Both of these two LA schemes broke the records of convergence rate when proposed, which confirmed the defect of traditional MLE.
2. On the other hand, there are attempts from the Bayesian perspective. Historically, one of the major reasons for avoiding Bayesian inference is that it can be computationally intensive under many circumstances. The rapid improvements in available computing power over the past few decades can, however, help overcome this obstacle, and Bayesian techniques are becoming more widespread not only in practical statistical applications but also in theoretical approaches to modeling human cognition. In Bayesian statistics, parameter estimation involves placing a probability distribution over model parameters. Concerning LA, the posterior distribution of *c*_*i*_ with respect to observed data is a beta distribution.
  In Zhang et al. (2013), DBPA was proposed where the posterior distribution of estimated $\hat{c_{i}}$ is represented by a beta distribution *Beta*(α, β), the parameter α and β record the number of times that a specific action has been rewarded and penalized, respectively. Then the 95^th^ percentile of the cumulative posterior distribution is utilized as an estimation of *c*_*i*_.

One of the main drawbacks of the way that information is being used by existing LA schemes is that they summarize beliefs about *c*_*i*_, such as the likelihood function or the posterior distribution, into a point estimate, which obviously may lead to information loss. In the proposed PFLA, we insist on taking advantage of the entire Bayesian posterior distribution of *c*_*i*_ for further statistical inference.

### 2.2. Optimal assertion

For the second challenge, as the collected information accumulates, we become more and more confident to make an assertion. But when is the exact timing?

The quantity of samples before the convergence of existing strategies is indirectly controlled by its learning parameters. Actually, the LA is not aware of whether it has collected enough information or not, as a consequence, its performance completely relies on the manual configuration of learning parameters inevitably. As far as we're concerned, there is no report describing a parameter-free scheme for learning in multi-action environments, and this research area remains quite open.

However, there are efforts from other research areas that shed some light on this target. In Granmo (2010), a Bayesian learning automaton (BLA) was proposed for solving the two-armed Bernoulli bandit (TABB) problem. The TABB problem is a classic optimization problem that explores the trade-off between exploitation and exploration in reinforcement learning. One distinct difference between learning automata and bandit-playing algorithms is the metrics used for performance evaluation. Typically, *accuracy* is used for evaluating LA algorithms while *regret* is usually used in bandit playing algorithms. Despite being presented with different objectives, BLA is somewhat related to our study and inspired our work. Therefore, the philosophy of BLA is briefly summarized as follows: The BLA maintains two beta distributions as estimates of the reward probabilities for the two arms (corresponding to actions in the LA field). At each time instance, two values are randomly drawn from the two beta distributions, respectively. The arm with the higher random value is selected, and the feedback is utilized to update the parameter of the beta distribution associated with the selected arm. One advantage of BLA is that it doesn't involve any explicit computation of Bayesian expression. In Granmo (2010), it has been claimed that BLA performs better than UCB-tuned, the best performing algorithm reported in Auer et al. (2002).

Inspired by Granmo (2010), we constructed the PFLA by using Bayesian inference to enable convergence self-judgment in this paper. In contrast to Granmo (2010), however, the probability of each arm being selected must be explicitly computed to judge the convergence of the algorithm. In addition, due to the poor performance of probability matching, we developed a deterministic exploration strategy. The technical details are provided in the next section.

## 3. A parameter-free learning automaton

In this section, we introduce each essential mechanism of our scheme in detail.

### 3.1. Self-judgment

Consider a P-model environment with *r* available actions, as we have no prior knowledge about these actions, each of them is possible to be the optimal one. We refer to these *r* possibilities as *r* hypotheses $H_{1},H_{2},\ldots ,H_{r}$ so that each hypothesis $H_{i}$ represents the event that action *a*_*i*_ is the optimal action.

As we discussed in Section 2, the Bayesian estimates of each action's reward probability just intuitively are beta distributed random variables, denoted as *E* = {*e*_1_, *e*_2_, …, *e*_*r*_}, where *e*_*i*_ ~ *Beta*(α_*i*_, β_*i*_).

Because the propositions $H_{1},H_{2},\ldots ,H_{r}$ are mutually exclusive and collectively exhaustive, apparently we have $\underset{i}{\sum }Pr\left(H_{i}\right)=1$. Therefore, we can simply assert that α_*i*_ is the optimal action once $Pr\left(H_{i}\right)$ is greater than some predefined threshold η. For this reason, the explicit computation of $Pr\left(H_{i}\right)$ is necessary here to make that assertion.

#### 3.1.1. Two-action environments

In the two-action case, $Pr\left(H_{1}\right)$ can be formulated in the following equivalent forms:

(1) $$\begin{array}{l} Pr\left(H_{1}\right)=Pr\left(e_{1}>e_{2}\right) \end{array}$$

(2) $$\begin{array}{l} =\overset{\alpha_{1}-1}{\underset{i=0}{\sum }}\frac{B\left(\alpha_{2}+i,\beta_{1}+\beta_{2}\right)}{\left(\beta_{1}+i\right)B\left(1+i,\beta_{1}\right)B\left(\alpha_{2},\beta_{2}\right)} \end{array}$$

(3) $$\begin{array}{l} =\overset{\beta_{2}-1}{\underset{i=0}{\sum }}\frac{B\left(\beta_{1}+i,\alpha_{1}+\alpha_{2}\right)}{\left(\alpha_{2}+i\right)B\left(1+i,\alpha_{2}\right)B\left(\alpha_{1},\beta_{1}\right)} \end{array}$$

(4) $$\begin{array}{l} =1-Pr\left(H_{2}\right) \end{array}$$

(5) $$\begin{array}{l} =1-\overset{\alpha_{2}-1}{\underset{i=0}{\sum }}\frac{B\left(\alpha_{1}+i,\beta_{1}+\beta_{2}\right)}{\left(\beta_{2}+i\right)B\left(1+i,\beta_{2}\right)B\left(\alpha_{1},\beta_{1}\right)} \end{array}$$

(6) $$\begin{array}{l} =1-\overset{\beta_{1}-1}{\underset{i=0}{\sum }}\frac{B\left(\beta_{2}+i,\alpha_{1}+\alpha_{2}\right)}{\left(\alpha_{1}+i\right)B\left(1+i,\alpha_{1}\right)B\left(\alpha_{2},\beta_{2}\right)} \end{array}$$

The above formulas can be easily implemented by a programming language with a well-defined log-beta function, thus the exact calculation of $Pr\left(H_{1}\right)$ can be completed within $O\left(\min\left(\alpha_{1},\alpha_{2},\beta_{1},\beta_{2}\right)\right)$. However, in multi-action cases, the closed-form of $Pr\left(H_{i}\right)$ is too complex and it's somewhat computationally intensive to calculate it directly. So in our scheme, a Monte Carlo simulation is adopted for evaluating $Pr\left(H_{i}\right)$ in a multi-action environment.

#### 3.1.2. Multi-action environments

The closed-form calculation of $Pr\left(H_{i}\right)$ is feasible for a small action set, but it becomes much more difficult as the number of actions increases.

Monte Carlo methods are a broad class of computational algorithms that rely on repeated random sampling to obtain numerical results.

In multi-action environments, in order to evaluate $Pr\left(H_{i}\right)$, an intuitive approach is to generate random samples from the *r* beta distributions and count how often the sample from *Beta*(α_*i*_, β_*i*_) is bigger than any other samples. In that way, the following Monte-Carlo simulation procedure is proposed.

Suppose the number of simulation replications is *N*. Since *e*_*i*_ follows *Beta*(α_*i*_, β_*i*_), let $x_{i}^{n}$ be one of the *r* random samples at the *n*th replication.

Then, $Pr\left(H_{i}\right)$ can be simulated as

(7) $$\begin{array}{l} \hat{Pr}\left(H_{i}\right)=\frac{1}{N}\overset{N}{\underset{n=1}{\sum }}I\left(x_{i}^{n}\right) \end{array}$$

where $I\left(x_{i}^{n}\right)$ is an indicator function such that

(8a)(8b) $$I(x_{i}^{n})=\left\{ \begin{array}{l} \begin{array}{cc} 1 & \text{if }x_{i}^{n}>x_{j}^{n},\forall j\neq i \end{array}\;\\ \begin{array}{cc} 0 & \text{otherwise} \end{array}\; \end{array}\right.$$

It is simple to verify that $\underset{i}{\sum }Pr\left(H_{i}\right)=1$.

### 3.2. Exploration strategy

In conventional estimator-based learning schemes, which are the majority family of LA, a stochastic exploration strategy is employed. A probability vector for choosing each action is maintained in the automaton and is properly updated under the guidance of the estimator and environment feedback after every interaction. However, such a probability vector does not exist in our scheme. Instead, a vector of probabilities indicating the chance of each action being the best one is maintained in our scheme. The exploration strategy in Granmo (2010) is the so-called *probability matching*, which occurs when an action is chosen with a frequency equivalent to the probability of that action being the best choice. In Ge et al. (2015b), we constructed a learning automata by adding an absorbing barrier to BLA and applying it as a baseline for comparison. The numerical simulation shows the low performance of the probability matching strategy in designing parameter-free LA. Therefore, a novel deterministic exploration strategy is proposed accordingly to overcome this pitfall.

Because $\max\left\{Pr\left(H_{i}\right)\right\}>\eta$ is the stop criterion of our scheme, in order to pursue a rapid convergence, one straightforward and obvious approach is maximizing the expected increment of $\max\left\{Pr\left(H_{i}\right)\right\}$ over the action set.

#### 3.2.1. Two-action environments

In two-action environments, if $Pr\left(H_{1}\right)$ is greater than $Pr\left(H_{2}\right)$, then we suppose action *a*_1_ is more likely to be the optimal one, and thus attempt to find out the action that will lead to the maximal expected increment of $Pr\left(H_{1}\right)$, or vice versa.

We denote $Pr\left(H_{1}\right)$ as *g*(α_1_, β_1_, α_2_, β_2_), and the following recurrence relations are derived (Cook, 2005):

(9) $$\begin{array}{l} g\left(\alpha_{1}+1,\beta_{1},\alpha_{2},\beta_{2}\right)=g\left(\alpha_{1},\beta_{1},\alpha_{2},\beta_{2}\right)+h\left(\alpha_{1},\beta_{1},\alpha_{2},\beta_{2}\right)/\alpha_{1} \end{array}$$

(10) $$\begin{array}{l} g\left(\alpha_{1},\beta_{1}+1,\alpha_{2},\beta_{2}\right)=g\left(\alpha_{1},\beta_{1},\alpha_{2},\beta_{2}\right)-h\left(\alpha_{1},\beta_{1},\alpha_{2},\beta_{2}\right)/\beta_{1} \end{array}$$

(11) $$\begin{array}{l} g\left(\alpha_{1},\beta_{1},\alpha_{2}+1,\beta_{2}\right)=g\left(\alpha_{1},\beta_{1},\alpha_{2},\beta_{2}\right)-h\left(\alpha_{1},\beta_{1},\alpha_{2},\beta_{2}\right)/\alpha_{2} \end{array}$$

(12) $$\begin{array}{l} g\left(\alpha_{1},\beta_{1},\alpha_{2},\beta_{2}+1\right)=g\left(\alpha_{1},\beta_{1},\alpha_{2},\beta_{2}\right)+h\left(\alpha_{1},\beta_{1},\alpha_{2},\beta_{2}\right)/\beta_{2} \end{array}$$

where $h\left(\alpha_{1},\beta_{1},\alpha_{2},\beta_{2}\right)=\frac{B\left(\alpha_{1}+\alpha_{2},\beta_{1}+\beta_{2}\right)}{B\left(\alpha_{1},\beta_{1}\right)B\left(\alpha_{2},\beta_{2}\right)}$.

Hence, given that action *a*_1_ is chosen, the conditional expected increment of $Pr\left(H_{1}\right)$ is:

(13) $$\begin{array}{l} 𝔼\left[\Delta Pr\left(H_{1}\right)|a_{1}\text{is chosen}\right] \end{array}$$

(14) $$\begin{array}{l} =c_{1}\times h\left(\alpha_{1},\beta_{1},\alpha_{2},\beta_{2}\right)/\alpha_{1}-\left(1-c_{1}\right)\times h\left(\alpha_{1},\beta_{1},\alpha_{2},\beta_{2}\right)/\beta_{1} \end{array}$$

(15) $$\begin{array}{l} =h\left(\alpha_{1},\beta_{1},\alpha_{2},\beta_{2}\right)\left(c_{1}/\alpha_{1}-\left(1-c_{1}\right)/\beta_{1}\right) \end{array}$$

because *c*_1_ is unknown to us, we can approximate the above equation as

(16) $$\begin{array}{l} 𝔼\left[\Delta Pr\left(H_{1}\right)|a_{1}\text{is chosen}\right] \end{array}$$

(17) $$\begin{array}{l} \approx h\left(\alpha_{1},\beta_{1},\alpha_{2},\beta_{2}\right)\left(\frac{\alpha_{1}}{\alpha_{1}+\beta_{1}}/\alpha_{1}-\frac{\beta_{1}}{\alpha_{1}+\beta_{1}}/\beta_{1}\right) \end{array}$$

(18) $$\begin{array}{l} =0 \end{array}$$

In the same way, we have

(19) $$\begin{array}{l} 𝔼\left[\Delta Pr\left(H_{1}\right)|a_{2}\text{is chosen}\right]\approx 0 \end{array}$$

(18) and (19) indicate that no matter which action is picked, the expected difference of $\max\left\{Pr\left(H_{i}\right)\right\}$ will approximately be zero, which makes it difficult for us to make decisions.

Our solution is to select the action that gives the expected maximum possible increment to $\max\left\{Pr\left(H_{i}\right)\right\}$, as we did in Ge et al. (2015b). More specifically, if $Pr\left(H_{1}\right)$ is greater than $Pr\left(H_{2}\right)$, then we try to find out the action that could probably lead to the expected maximal increment of $Pr\left(H_{1}\right)$, that is

(20) $$\begin{array}{l} \underset{i}{\mathrm{argmax}}𝔼\left[\max\left\{\Delta Pr\left(H_{1}\right)\right\}|a_{i}\text{is chosen}\right] \end{array}$$

Otherwise, we try to maximize

(21) $$\begin{array}{l} \underset{i}{\mathrm{argmax}}𝔼\left[\max\left\{\Delta Pr\left(H_{2}\right)\right\}|a_{i}\text{is chosen}\right] \end{array}$$

The events that can lead to increments of $Pr\left(H_{1}\right)$ are “*action a*_1_ *is selected and rewarded*” and “*action a*_2_ *is selected and punished*.” Hence the optimization objective of (20) can be simplified as:

(22a)(22b) $$\{\begin{array}{ll} \frac{c_{1}}{\alpha_{1}}h(\alpha_{1},\beta_{1},\alpha_{2},\beta_{2}) & a_{1}\text{ is chosen}\\ \frac{1-c_{2}}{\beta_{2}}h(\alpha_{1},\beta_{1},\alpha_{2},\beta_{2}) & a_{2}\text{ is chosen} \end{array}$$

By employing the Maximum Likelihood Estimate of *c*_1_ and *c*_2_, (22) can be written as

(23a)(23b) $$\begin{array}{l} \{\begin{array}{ll} \frac{h(\alpha_{1},\beta_{1},\alpha_{2},\beta_{2})}{\left(\alpha_{1}+\beta_{1}\right)} & a_{1}\text{ is chosen}\\ \frac{h(\alpha_{1},\beta_{1},\alpha_{2},\beta_{2})}{\left(\alpha_{2}+\beta_{2}\right)} & a_{2}\text{ is chosen} \end{array} \end{array}$$

The same conclusion holds also for situation $Pr\left(H_{1}\right)<Pr\left(H_{2}\right)$.

As a result, the strategy adopted in two-action environments is selecting the action which has been observed less between the two candidate actions at every time instance, as (24) reveals.

(24a)(24b) $$\{\begin{array}{ll} \underset{i}{\text{argmin}}(\alpha_{i}+\beta_{i}) & \text{when }S_{1}\neq S_{2}\\ \text{randomly chosen} & \text{when }S_{1}=S_{2} \end{array}$$

#### 3.2.2. Multi-action environments

In multi-action environments, the automaton has to distinguish the best action from the action set. Intuitively, we can maximize the expected increment of $Pr\left(H_{i}\right)$ over the selection of actions, however, the closed form of $Pr\left(H_{i}\right)$ is complicated, making the exact solution computationally intractable.

However, from an alternative perspective, the automaton only needs to determine which is the best of the top two possibly optimal actions. That is, for the two actions which are most possible to be the optimal action, denoted as action *a*_*i*1_ and action *a*_*i*2_, we only have to maximize the probability *Pr*(*e*_*i*1_ > *e*_*i*2_) or *Pr*(*e*_*i*2_ > *e*_*i*1_), exactly the same as it in two-action environments. So we come to the conclusion that, in the proposed scheme, our exploration strategy is similar to (24).

### 3.3. Initialization of beta distributions

In our scheme, each estimation *e*_*i*_ is represented by a beta distribution *e*_*i*_ ~ *Beta*(α_*i*_, β_*i*_). The parameters α_*i*_ and β_*i*_ record the number of times that action *a*_*i*_ has been rewarded and punished, respectively.

In the beginning, as we know nothing about the actions, a non-informative (uniform) prior distribution is advised to infer the posterior distribution. So α_*i*_ and β_*i*_ should be set identically to 1, exactly the same as in Granmo (2010) and Zhang et al. (2013).

However, as clarified in Sutton and Barto (2018), initial action values can be used as a simple way of encouraging exploration. The technique of *optimistic initial values* is applied, which has been reported as a quite effective simple trick on stationary problems.

Therefore, in our scheme, the prior distribution is *Beta*(2, 1) for inferring the posterior distribution, i.e., all beta random variables are initialized as α_*i*_ = 2, β_*i*_ = 1.

The estimates of all actions' reward probability are intentionally biased toward 1. The impact of the bias is permanent, though decreasing over iterations. When an action has been sampled just a few times, the bias contributes a large proportion to the estimate, thus further exploration is encouraged. By the time an action has been observed many times, the impact of the biased initial value is negligible.

Finally, the overall process of PFLA is summarized in Algorithm 1.

**Algorithm 1:** Parameter-free learning automaton.

**Require:** η: a convergence threshold; *N*: the number of replications of Monte Carlo simulation
1: **Initial** α_*i*_ = 2, β_*i*_ = 1 for *i* = 1, 2, 3, …, *r*;
2: **repeat**
3: Evaluate the probability $\hat{Pr}\left(H_{i}\right)$ according to (7) for each action *i* = 1, 2, 3, …, *r*;
4: Choose the two actions with top two $\hat{Pr}\left(H_{i}\right)$, denoted as *a*_*i*1_ and *a*_*i*2_. If there are two or more actions with identical maximum $\hat{Pr}\left(H_{i}\right)$, then choose two from them randomly.
5: Select one from *a*_*i*1_ and *a*_*i*2_ according to $$\begin{array}{l} \{\begin{array}{cc} a_{i}=a_{i1} & \text{if }S_{i1}<S_{i2}\\ a_{i2} & \text{if }S_{i1}>S_{i2}\\ \text{randomly chosen} & \text{if }S_{i1}=S_{i2} \end{array} \end{array}$$ and interacts with the environment.
6: Receive feedback from the environment and update the parameters of beta distributions for action *a*_*i*_: $$\begin{array}{l} \{\begin{array}{cc} \alpha_{i}=\alpha_{i}+1 & \text{if a reward is received}\\ \beta_{i}=\beta_{i}+1 & \text{if a penalty is received} \end{array} \end{array}$$
7: **until** $\max\left\{\hat{Pr}\left(H_{i}\right)\right\}>\eta$

## 4. Performance analysis

In this section, the statistical performance of the proposed scheme is analyzed, an approximate lower bound of the accuracy is derived and the ϵ-optimality of the proposed scheme is further proved.

### 4.1. An approximate lower bound of the accuracy

As declared in Owen (2013), from the central limit theorem (CLT), we know that the error of Monte Carlo simulation has approximately a normal distribution with zero mean and variance σ^2^/*N*. Hence, if we denote the error between $Pr\left(H_{i}\right)$ and its Monte-Carlo estimate as ϵ_*i*_, then we get

(27) $$\begin{array}{l} Pr\left(H_{i}\right)=\hat{Pr}\left(H_{i}\right)+\epsilon_{i} \end{array}$$

(28) $$\begin{array}{l} \geq \eta +\epsilon_{i} \end{array}$$

(29) $$\begin{array}{l} \sim \eta +Norm\left(0,\frac{\sigma_{i}^{2}}{N}\right) \end{array}$$

(30) $$\begin{array}{l} \geq \eta -|\epsilon_{i}| \end{array}$$

where $\hat{Pr}\left(H_{i}\right)$ is the Monte-Carlo estimate of $Pr\left(H_{i}\right)$ and $\sigma_{i}^{2}$ is the variance of *I*(*x*_*i*_).

We may note that the right-hand side of (29) is irrelevant to the characteristics of the environment. In other words, the performance of the proposed scheme only depends on the selection of η and *N*. That is the theoretical foundation of the parameter-free property.

**Figure 2:**
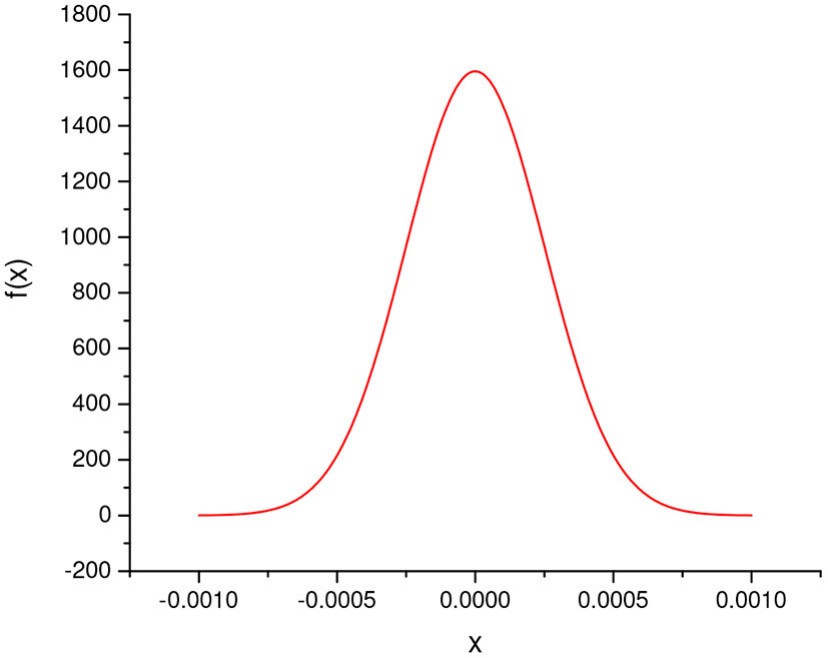
The probability density function of $Norm\left(0,\frac{1}{4,000}\right)$.

As the outcome of *I*(*x*_*i*_) is binary, in the worst case, the maximum of $\sigma_{i}^{2}$ is 0.25. When *N* equals 1,000, the probability density function of ϵ_*i*_ is shown in Figure 2, which quantitatively depicts the error. Obviously, the error is so small that could be ignored.

Therefore, the approximate lower bound of $Pr\left(H_{i}\right)$ is η. According to the Bayesian theory, the accuracy of our scheme is approximately larger than η.

Next, we shall describe the behavior of the proposed scheme more precisely. Like the pioneers have done in previous literature, the ϵ-optimality of the proposed scheme will be derived.

### 4.2. Proof of ε-optimality

Recall that *e*_*i*_ is defined as the estimated reward probability of action *a*_*i*_ and follows *Beta*(α_*i*_, β_*i*_), which is the posterior distribution of the estimated reward probability. The probability density function of *Beta*(α_*i*_, β_*i*_) is $f\left(x_{i};\alpha_{i},\beta_{i}\right)=C_{i}x_{i}^{\alpha_{i}-1}{\left(1-x_{i}\right)}^{\beta_{i}-1}$, where $C_{i}=\frac{1}{B\left(\alpha_{i},\beta_{i}\right)}$ serves as a normalizing factor such that $\int_{0}^{1}f\left(x_{i};\alpha_{i},\beta_{i}\right)=1$. Let *Z*_*i*_ = α_*i*_ − 2 and *W*_*i*_ = β_*i*_ − 1 denote the numbers of times that action *a*_*i*_ has been rewarded and penalized, respectively, and *S*_*i*_ = *Z*_*i*_ + *W*_*i*_ = α_*i*_ + β_*i*_ − 3 be the number of times that action *a*_*i*_ has been selected.

Based on these preliminaries, the following Lemmas and Theorems are proposed:

**Lemma 1**. *The beta distribution *Beta*(α_*i*_, β_*i*_) becomes 1-point Degenerate distribution with a Dirac delta function spike at *c*_*i*_, provided that the number of selecting action *a*_*i*_ approaches infinity, i.e.,* ∀*ε* > 0,

(31) $$\begin{array}{l} \underset{S_{i}\to \infty }{\lim}\int_{|x_{i}-c_{i}|\leq \varepsilon ⋂\left[0,1\right]}f\left(x_{i};\alpha_{i},\beta_{i}\right)dx_{i}=1 \end{array}$$

(32) $$\begin{array}{l} \underset{S_{i}\to \infty }{\lim}\int_{|x_{i}-c_{i}|>\varepsilon ⋂\left[0,1\right]}f\left(x_{i};\alpha_{i},\beta_{i}\right)dx_{i}=0 \end{array}$$

**Proof 1**. *According to the law of large numbers, we have* $\frac{Z_{i}}{S_{i}}\to c_{i}$, *as S*_*i*_ → ∞.

*Hence*

(33) $$\begin{array}{l} \begin{array}{l} \underset{S_{i}\to \infty }{\lim}\frac{\alpha_{i}-1}{S_{i}}=\frac{Z_{i}+1}{S_{i}}=c_{i}\\ \underset{S_{i}\to \infty }{\lim}\frac{\beta_{i}-1}{S_{i}}=\frac{S_{i}-Z_{i}}{S_{i}}=\left(1-c_{i}\right) \end{array}\}\Rightarrow \{\begin{array}{l} \alpha_{i}-1=c_{i}S_{i}\\ \beta_{i}-1=\left(1-c_{i}\right)S_{i} \end{array} \end{array}$$

*The probability density function takes the form:*

(34) $$\begin{array}{l} \underset{S_{i}\to \infty }{\lim}f\left(x_{i};\alpha_{i},\beta_{i}\right)=C_{i}x_{i}^{\alpha_{i}-1}{\left(1-x_{i}\right)}^{\beta_{i}-1} \end{array}$$

(35) $$\begin{array}{l} =C_{i}{\left[x_{i}^{c_{i}}{\left(1-x_{i}\right)}^{1-c_{i}}\right]}^{S_{i}} \end{array}$$

(36) $$\begin{array}{l} =C_{i}g^{S_{i}}\left(x_{i}\right) \end{array}$$

*where* $g\left(x_{i}\right)=x_{i}^{c_{i}}{\left(1-x_{i}\right)}^{1-c_{i}}$.

*Note that g*(*x*_*i*_) *is a non-negative integrable function, we have*

(37) $$\begin{array}{l} \underset{S_{i}\to \infty }{\lim}{\left(\int_{0}^{1}g^{S_{i}}\left(x_{i}\right)\right)}^{\frac{1}{S_{i}}}dx_{i}=||g|{|}_{\infty }. \end{array}$$

*Therefore,*

(38) $$\begin{array}{l} \underset{S_{i}\to \infty }{\lim}C_{i}^{\frac{1}{S_{i}}}=\frac{1}{{\left(\int_{0}^{1}g^{S_{i}}\left(x_{i}\right)dx_{i}\right)}^{\frac{1}{S_{i}}}}=\frac{1}{||g|{|}_{\infty }} \end{array}$$

*This reveals, as S*_*i*_ → ∞

(39) $$\begin{array}{l} {\left(\int_{|x_{i}-c_{i}|>\varepsilon ⋂\left[0,1\right]}f\left(x_{i};\alpha_{i},\beta_{i}\right)dx_{i}\right)}^{\frac{1}{S_{i}}}\\ =C_{i}^{\frac{1}{S_{i}}}{\left(\int_{|x_{i}-c_{i}|>\varepsilon ⋂\left[0,1\right]}g^{S_{i}}\left(x_{i}\right)dx_{i}\right)}^{\frac{1}{S_{i}}}\to \frac{||g|{|}_{\infty ,\varepsilon }}{||g|{|}_{\infty }} \end{array}$$

*where* ||*g*||_∞,ε_ *is the L*^∞^ *norm of g when restricted to* |*x*_*i*_ − *c*_*i*_| > ε.

*By taking both sides of (39) to the S*_*i*_ *power, we obtain*

(40) $$\begin{array}{l} \int_{|x_{i}-c_{i}|>\varepsilon ⋂\left[0,1\right]}f\left(x_{i};\alpha_{i},\beta_{i}\right)dx_{i}\to {\left(\frac{||g|{|}_{\infty ,\varepsilon }}{||g|{|}_{\infty }}\right)}^{S_{i}} \end{array}$$

*Obviously* $\frac{||g|{|}_{\infty ,\varepsilon }}{||g|{|}_{\infty }}<1$, *for the fact that g is continuous and has a unique maximum at c*_*i*_, *thus*

(41) $$\begin{array}{l} \int_{|x_{i}-c_{i}|>\varepsilon ⋂\left[0,1\right]}f\left(x_{i};\alpha_{i},\beta_{i}\right)dx_{i}\to 0 \end{array}$$

*as S*_*i*_ → ∞.

*Note that* $\int_{0}^{1}f\left(x_{i};\alpha_{i},\beta_{i}\right)dx_{i}=1$ *and the proof is finished*.

**Lemma 2**. *For two or more random variables e*_*i*_ ~ *Beta*(*α*_*i*_, *β*_*i*_), *assume m is the index of action that has the maximum reward probability such that c*_*m*_ = max(*c*_*i*_), *then*

(42) $$\begin{array}{l} \underset{S_{i}\to \infty }{\lim}Pr\left\{e_{m}>\underset{i\neq m}{\max}\left(e_{i}\right)\right\}=1 \end{array}$$

**Proof 2**.

(43) $$\begin{array}{l} Pr\left\{e_{m}>\underset{i\neq m}{\max}\left(e_{i}\right)\right\}\\ =\int_{0}^{1}f\left(x_{m};\alpha_{m},\beta_{m}\right)\underset{i\neq m}{\prod }\left[\int_{0}^{x_{m}}f\left(x_{i};\alpha_{i},\beta_{i}\right)dx_{i}\right]dx_{m} \end{array}$$

*From Lemma 1, we know that f*(*x*_*i*_; *α*_*i*_, *β*_*i*_) → *δ*(*x*_*i*_ − *c*_*i*_) *as S*_*i*_ → ∞.

*By using the sampling property of the Dirac delta function, (43) can be simplified as*

(44) $$\begin{array}{l} \underset{S_{i}\to \infty }{\lim}\mathrm{Pr}\left\{e_{m}>\underset{i\neq m}{\max}\left(e_{i}\right)\right\}=\underset{S_{i}\to \infty }{\lim}\underset{i\neq m}{\prod }\int_{0}^{c_{m}}f\left(x_{i};\alpha_{i},\beta_{i}\right)dx_{i} \end{array}$$

(45) $$\begin{array}{l} =\underset{S_{i}\to \infty }{\lim}\underset{i\neq m}{\prod }\int_{0}^{c_{m}}\delta \left(x_{i}-c_{i}\right)dx_{i} \end{array}$$

*Note that* ∀*i* ≠ *m*, *as c*_*i*_ ∈ [0, *c*_*m*_], $\int_{0}^{c_{m}}\delta \left(x_{i}-c_{i}\right)dx_{i}=1$. *And finally*

(46) $$\begin{array}{l} \underset{S_{i}\to \infty }{\lim}Pr\left\{e_{m}>\underset{i\neq m}{\max}\left(e_{i}\right)\right\}=1 \end{array}$$

*This completes the proof*.

**Remark 1**. *It is noted that, Lemma 2 implies* $\lim_{S_{i}\to \infty }Pr\left\{H_{m}\right\}=1$

**Lemma 3**. *Suppose one component of the vector* $\left\{Pr\left(H_{1}\right),Pr\left(H_{2}\right),\ldots ,Pr\left(H_{r}\right)\right\}$, *say* $Pr\left(H_{i}\right)$ *approaches 1 only if the number of each action been selected S*_*i*_ → ∞, *for all i* ∈ {1, 2, …, *r*}.

**Proof 3**. *As* $Pr\left(H_{i}\right)\to 1$, *for any* δ > 0, *we have* $Pr\left(H_{i}\right)\geq 1-\delta$, *hence*

(47) $$\begin{array}{l} Pr\left(H_{i}\right)=\int_{0}^{1}f\left(x_{i};\alpha_{i},\beta_{i}\right)\underset{j\neq i}{\prod }\left[\int_{0}^{x_{i}}f\left(x_{j};\alpha_{j},\beta_{j}\right)dx_{j}\right]dx_{i} \end{array}$$

(48) $$\begin{array}{l} =\int_{0}^{1}f\left(x_{i};\alpha_{i},\beta_{i}\right)\int_{0}^{x_{i}}f\left(x_{j};\alpha_{j},\beta_{j}\right)dx_{j}\\ \underset{k\neq i,k\neq j}{\prod }\left[\int_{0}^{x_{i}}f\left(x_{k};\alpha_{k},\beta_{k}\right)dx_{k}\right]dx_{i} \end{array}$$

(49) $$\begin{array}{l} \leq \int_{0}^{1}f\left(x_{i};\alpha_{i},\beta_{i}\right)\int_{0}^{x_{i}}f\left(x_{j};\alpha_{j},\beta_{j}\right)dx_{j}\\ \underset{k\neq i,k\neq j}{\prod }\left[\int_{0}^{1}f\left(x_{k};\alpha_{k},\beta_{k}\right)dx_{k}\right]dx_{i} \end{array}$$

(50) $$\begin{array}{l} =\int_{0}^{1}f\left(x_{i};\alpha_{i},\beta_{i}\right)\int_{0}^{x_{i}}f\left(x_{j};\alpha_{j},\beta_{j}\right)dx_{j}dx_{i} \end{array}$$

(51) $$\begin{array}{l} =Pr\left\{e_{i}>e_{j}\right\} \end{array}$$

*As a result, for all j* ≠ *i*,

(52) $$\begin{array}{l} Pr\left\{e_{i}>e_{j}\right\}\geq Pr\left(H_{i}\right)\to 1 \end{array}$$

(53) $$\begin{array}{l} \Rightarrow Pr\left\{e_{j}>e_{i}\right\}\to 0 \end{array}$$

(54) $$\begin{array}{l} \Rightarrow \int_{0}^{1}f\left(x_{j};\alpha_{j},\beta_{j}\right)\int_{0}^{x_{j}}f\left(x_{i};\alpha_{i},\beta_{i}\right)dx_{i}dx_{j}\to 0 \end{array}$$

*By denoting* $F\left(x\right)=f\left(x;\alpha_{j},\beta_{j}\right)B\left(x;\alpha_{i},\beta_{i}\right)=f\left(x;\alpha_{j},\beta_{j}\right)\int_{0}^{x}f\left(x_{i};\alpha_{i},\beta_{i}\right)dx_{i}$, *we have*

(55) $$\begin{array}{l} \int_{0}^{1}F\left(x\right)dx\to 0 \end{array}$$

*Suppose at least one of S*_*i*_ *and S*_*j*_ *is not infinity, thus three possible cases should be discussed*.

1. Case *S*_*i*_ < ∞ and *S*_*j*_ < ∞.
  *In this case, f*(*x*_*j*_; *α*_*j*_, *β*_*j*_) *is a continuous function and strictly positive on* (0, 1). *As* $\frac{dB\left(x;\alpha_{i},\beta_{i}\right)}{dx}=f\left(x_{i};\alpha_{i},\beta_{i}\right)$ *is continuous, B*(*x*; α_*i*_, *β*_*i*_) *is continuously differentiable which implies it is a continuous function. In addition, B*(*x*; α_*i*_, *β*_*i*_) *is strictly positive on* (0, 1). *Clearly, the product of two strictly positive continuous functions F*(*x*) *is continuous and F*(*x*) > 0 *on the interval* (0, 1), *hence*
  (56) $$\begin{array}{l} \int_{0}^{1}F\left(x\right)dx>0 \end{array}$$
  *which contradicts (55)*.
2. *Case S*_*i*_ < ∞ *and S*_*j*_ = ∞.
  *Similarly, we can prove that B*(*x*; α_*i*_, *β*_*i*_) *is strictly positive and continuous on* (0, 1), *and f*(*x*; α_*j*_, *β*_*j*_) → δ(*x* − *c*_*j*_).
  *Hence, (54) can be written as:*
  (57) $$\begin{array}{l} B\left(c_{j};\alpha_{i},\beta_{i}\right)\to 0 \end{array}$$
  *that contradicts the fact that B*(*x*; α_*i*_, *β*_*i*_) *is strictly positive on* (0, 1).
3. *Case S*_*i*_ = ∞ and *S*_*j*_ < ∞.
  *Similarly, we can prove that f*(*x*_*j*_; *α*_*j*_, *β*_*j*_) *is strictly positive and continuous on* (0, 1), *and f*(*x*_*i*_; *α*_*i*_, *β*_*i*_) → δ(*x* − *c*_*i*_).
  *Hence, (54) can be written as:*
  (58) $$\begin{array}{l} \int_{c_{i}}^{1}f\left(x;\alpha_{j},\beta_{j}\right)dx\to 0 \end{array}$$
  *which implies f*(*x*_*j*_; *α*_*j*_, *β*_*j*_) = 0 *on* (*c*_*i*_, 1), *that contradicts the fact that f*(*x*_*j*_; *α*_*j*_, *β*_*j*_) *is strictly positive on* (0, 1).

*By summarizing the above three cases, we conclude that the supposition is false and both S*_*i*_ *and S*_*j*_ *must be infinity*.

*As i, j enumerate all the action indexes, the proof is completed*.

**Remark 2**. *From Lemma 3 and Remark 1, one can immediately see that given a threshold η* → 1, *PFLA will converge to the optimal action w.p.1 whenever it gets converged*.

**Lemma 4**. *The Monte Carlo estimation of* $Pr\left(H_{i}\right)$ *will converge almost surely as the number of Monte Carlo replications N tends to infinity, i.e.,*

(59) $$\begin{array}{l} Pr\left\{\underset{N\to \infty }{\lim}\hat{Pr}\left(H_{i}\right)=Pr\left(H_{i}\right)\right\}=1 \end{array}$$

**Proof 4**. *This lemma can be easily derived according to the strong law of large numbers*.

Let us now state and prove the main result for algorithm PFLA.

**Theorem 1**. *PFLA is ϵ-optimal in every stationary random environment. That is, given any ε* > 0, *there exists a N*_0_ < ∞, *a t*_0_ < ∞, *and a η*_0_ < 1 *such that for all t* ≥ *t*_0_, *N* ≥ *N*_0_ *and η > η*_0_:

(60) $$\begin{array}{l} \hat{Pr}\left(H_{m}\right)>1-\varepsilon \end{array}$$

**Proof 5**. *The theorem is equivalent to showing that,*

(61) $$\begin{array}{l} Pr\left\{\underset{\begin{array}{c} N\to \infty \\ t\to \infty \\ \eta \to 1 \end{array}}{\lim}\hat{Pr}\left(H_{m}\right)=1\right\}=1 \end{array}$$

*From Lemma 4, we know that (61) is equivalent to*

(62) $$\begin{array}{l} Pr\left\{\underset{\begin{array}{c} t\to \infty \\ \eta \to 1 \end{array}}{\lim}Pr\left(H_{m}\right)=1\right\}=1 \end{array}$$

*And according to Remark 2, we only need to prove that the scheme can definitely get converged, i.e., at least one of the components* $\left\{Pr\left(H_{1}\right),Pr\left(H_{2}\right),\ldots ,Pr\left(H_{r}\right)\right\}$ *approaches 1, as t* → ∞ *and η* → 1.

*Suppose the scheme has not converged yet at time t*_1_, *because exactly one action will be explored at each time instant, we have* $\underset{i}{\sum }S_{i}=t_{1}$.

*As t*_1_ → ∞, *a finite series has an infinite sum, which indicates that at least one of the terms *S*_*i*_ has an infinite value*.

*Then denote the set of actions, whose corresponding observation times S*_*i*_(*t*_1_) → ∞, *as* 𝔸_1_, *and denote the absolute complement set of* 𝔸_1_ *as* 𝔸_2_.

1. *If* 𝔸_2_ = ∅, *then for any action a*_*i*_, *we have S*_*i*_ → ∞.
  *By considering Remark 1, we have*
  (63) $$\begin{array}{l} Pr\left(H_{m}\right)\to 1 \end{array}$$
2. *We will show that if* 𝔸_2_ ≠ ∅, *then it is impossible that both the top two possibly optimal actions belong to set* 𝔸_1_.
  *Denote the action in* 𝔸_1_ *with the highest reward probability as a*_*m*1_, *then according to Lemma 2*, ∀*a*_*i*_ ∈ 𝔸_1_ and *i* ≠ *m*1,
  (64) $$\begin{array}{l} Pr\left(H_{i}\right)\to 0. \end{array}$$
  *While for actions a*_*j*_ ∈ 𝔸_2_,
  (65) $$\begin{array}{l} Pr\left(H_{j}\right)=\int_{0}^{1}f\left(x_{j};\alpha_{j},\beta_{j}\right)\underset{k\neq j}{\prod }\left[\int_{0}^{x_{j}}f\left(x_{k};\alpha_{k},\beta_{k}\right)dx_{k}\right]dx_{j} \end{array}$$
  (66) $$\begin{array}{l} =\int_{0}^{1}f\left(x_{j};\alpha_{j},\beta_{j}\right)\underset{a_{k1}\in {𝔸}_{1}}{\prod }\left[\int_{0}^{x_{j}}f\left(x_{k1};\alpha_{k1},\beta_{k1}\right)dx_{k1}\right]\\ \underset{k2\neq i,a_{k2}\in {𝔸}_{2}}{\prod }\left[\int_{0}^{x_{j}}f\left(x_{k2};\alpha_{k2},\beta_{k2}\right)dx_{k2}\right]dx_{j} \end{array}$$
  (67) $$\begin{array}{l} =\int_{0}^{1}f\left(x_{j};\alpha_{j},\beta_{j}\right)\underset{a_{k1}\in {𝔸}_{1}}{\prod }I\left(x_{j}\geq c_{k1}\right)\\ \underset{k2\neq i,a_{k2}\in {𝔸}_{2}}{\prod }\left[\int_{0}^{x_{j}}f\left(x_{k2};\alpha_{k2},\beta_{k2}\right)dx_{k2}\right]dx_{j} \end{array}$$
  (68) $$\begin{array}{l} =\int_{c_{m1}}^{1}f\left(x_{j};\alpha_{j},\beta_{j}\right)\\ \underset{k2\neq i,a_{k2}\in {𝔸}_{2}}{\prod }\left[\int_{0}^{x_{j}}f\left(x_{k2};\alpha_{k2},\beta_{k2}\right)dx_{k2}\right]dx_{j} \end{array}$$
  *As c*_*m*1_ < 1, *and the integrand is strictly positive and continuous. Obviously, (68) is larger than zero trivially*.
  *For actions in* 𝔸_1_ *other than a*_*m*1_, $Pr\left(H_{i}\right)\to 0$, *while for actions in* 𝔸_2_, *all* $Pr\left(H_{i}\right)$ *equal some constants that are larger than zero. Hence, at least one action of the top two most probably optimal actions is from* 𝔸_2_ *and this action will be chosen to draw feedback*.
  *As time t* → ∞, *once* 𝔸_2_ ≠ ∅, *one action in* 𝔸_2_ *will be explored. As a consequence, we can always find a t*_0_ > *t*_1_ *such that all actions in* 𝔸_2_ *will be explored infinite times and yield an empty* 𝔸_2_.

*Combining the above two cases, we may infer that all actions will be explored an infinite number of times and* $Pr\left(H_{m}\right)\to 1$.

*This completes the proof*.

## 5. Simulation results

During the last decade, SE_*ri*_ has been considered as the state-of-the-art algorithm for a long time, however, some recently proposed algorithms (Ge et al., 2015a; Jiang et al., 2015) claim a faster convergence than SE_*ri*_. To make a comprehensive comparison among currently available techniques, as well as to verify the effectiveness of the proposed parameter-free scheme, in this section, PFLA is compared with several classic parameter-based learning automata schemes, including DP_*ri*_ (Oommen and Lanctôt, 1990), DGPA (Agache and Oommen, 2002), DBPA (Zhang et al., 2013), DGCPA^*^ (Ge et al., 2015a), SE_*ri*_ (Papadimitriou et al., 2004), GBSE (Jiang et al., 2015), and LELA_*R*_ (Zhang et al., 2014).

All the schemes are evaluated in four two-action benchmark environments (Ge et al., 2015b) and five 10-action benchmark environments (Papadimitriou et al., 2004). The actions' reward probabilities for each environment are as follows:

*E*_1_ :{0.90, 0.60}
*E*_2_ :{0.80, 0.50}
*E*_3_ :{0.80, 0.60}
*E*_4_ :{0.20, 0.50}
*E*_5_ :{0.65, 0.50, 0.45, 0.40, 0.35, 0.30, 0.25, 0.20, 0.15, 0.10}
*E*_6_ :{0.60, 0.50, 0.45, 0.40, 0.35, 0.30, 0.25, 0.20, 0.15, 0.10}
*E*_7_ :{0.55, 0.50, 0.45, 0.40, 0.35, 0.30, 0.25, 0.20, 0.15, 0.10}
*E*_8_ :{0.70, 0.50, 0.30, 0.20, 0.40, 0.50, 0.40, 0.30, 0.50, 0.20}
*E*_9_ :{0.10, 0.45, 0.84, 0.76, 0.20, 0.40, 0.60, 0.70, 0.50, 0.30}

The comparison is organized in two ways:

1. Comparison between PFLA and parameter-based schemes with their learning parameters being carefully tuned.
2. Comparison between PFLA and parameter-based schemes without parameter tuning, using either pre-defined or randomly selected learning parameters.

### 5.1. Comparison with well-tuned schemes

Firstly, the parameter-based schemes are simulated with carefully tuned best parameters. The procedure for obtaining the best parameters is elaborated in the Appendix. The proposed PFLA, by contrast, takes identical parameter values of η = 0.99 and *N* = 1,000 in all nine environments.

The results of the simulations are summarized in Tables 2, 3. The *accuracy* is defined as *the ratio between the number of correct convergence and the number of experiments*, whilst the *iteration* as *the averaged number of required interactions between automaton and environment for a correct convergence*. It is noted that the initialization cost of estimators is also included. The number of initializations for each action is 10.

**Table 2:** Accuracy (*number of correct convergences/number of experiments*) of the compared algorithms in environments *E*_1_ to *E*_9_, when using the “best” learning parameters (250,000 experiments were performed for each scheme in each environment).

**Env**.	**DP_*ri*_**	**DGPA**	**DBPA**	**DGCPA**	**SE_*ri*_**	**GBSE**	**LELA_*R*_**	**PFLA**
*E*_1_	0.997	0.998	0.997	0.997	0.998	0.998	0.998	0.999
*E*_2_	0.997	0.997	0.998	0.998	0.997	0.998	0.998	0.999
*E*_3_	0.996	0.996	0.996	0.997	0.997	0.997	0.997	0.998
*E*_4_	0.998	0.997	0.998	0.997	0.998	0.998	0.998	0.999
*E*_5_	0.995	0.997	0.996	0.997	0.997	0.997	0.997	0.997
*E*_6_	0.994	0.996	0.994	0.996	0.996	0.996	0.996	0.999
*E*_7_	0.993	0.995	0.993	0.995	0.995	0.995	0.995	0.996
*E*_8_	0.996	0.997	0.996	0.998	0.998	0.998	0.997	0.999
*E*_9_	0.994	0.997	0.994	0.997	0.997	0.997	0.997	0.997

**Table 3:** Comparison of the average number of iterations required for convergence of the compared algorithms in environments *E*_1_ to *E*_9_.

**Env**.		***E*_1_**	***E*_2_**	***E*_3_**	***E*_4_**	***E*_5_**	***E*_6_**	***E*_7_**	***E*_8_**	***E*_9_**
DP_*ri*_	Para.^*a*^	*n* = 22	*n* = 29	*n* = 74	*n* = 18	*n* = 298	*n* = 653	*n* = 2,356	*n* = 216	*n* = 881
	Iter.^*b*^	46	61	127	64	1,086	2,500	9,613	783	2,363
DGPA	Para.	*n* = 20	*n* = 28	*n* = 70	*n* = 32	*n* = 33	*n* = 65	*n* = 204	*n* = 28	*n* = 55
	Iter.	52	66	141	72	880	1,677	5,191	754	1,445
DBPA	Para.	*n* = 20	*n* = 24	*n* = 57	*n* = 13	*n* = 102	*n* = 216	*n* = 820	*n* = 57	*n* = 326
	Iter.	44	54	105	52	646	1,419	5,423	432	1,384
DGCPA	Para.	(12, 1)	(18, 2)	(38, 3)	(18, 3)	(3, 5)	(6, 9)	(17, 20)	(2, 4)	(5, 7)
	Iter.	41	52	99	50	351	678	2032	298	598
SE_*ri*_	Para.	(15, 3)	(18, 3)	(38, 5)	(12, 3)	(16, 8)	(32, 12)	(105, 25)	(13, 6)	(33, 12)
	Iter.	43	51	99	54	426	834	2,540	325	729
GBSE	Para.	(12, 3)	(14, 3)	(22, 5)	(8, 3)	(1, 7)	(3, 9)	(6, 17)	(1, 5)	(3, 8)
	Iter.	43	53	97	55	401	772	2,262	306	612
LELA_*R*_	Para.	*n* = 13	*n* = 16	*n* = 41	*n* = 9	*n* = 9	*n* = 17	*n* = 59	*n* = 9	*n* = 24
	Iter.	51	65	137	63	629	1,129	3,733	586	1,072
PFLA	Iter.	44	51	102	54	510	934	2,737	538	735

For all schemes, the “best” learning parameters for each environment are used (250,000 experiments were performed for each scheme in each environment).

a Para., Parameter. And for methods that have more than one tunable parameter, a tuple is used to represent the parameters. For example, *n* = 38, γ = 5 is represented as (38, 5) in the table.

b Iter., Iteration.

In Table 2, PFLA converges with relatively high accuracy consistently, coinciding with our analytical results in Section 4, and verifying the effectiveness of our proposed parameter-free scheme. And since the accuracies of all schemes are close, their convergence rates can be “fairly” compared^4^.

**Figure 3:**
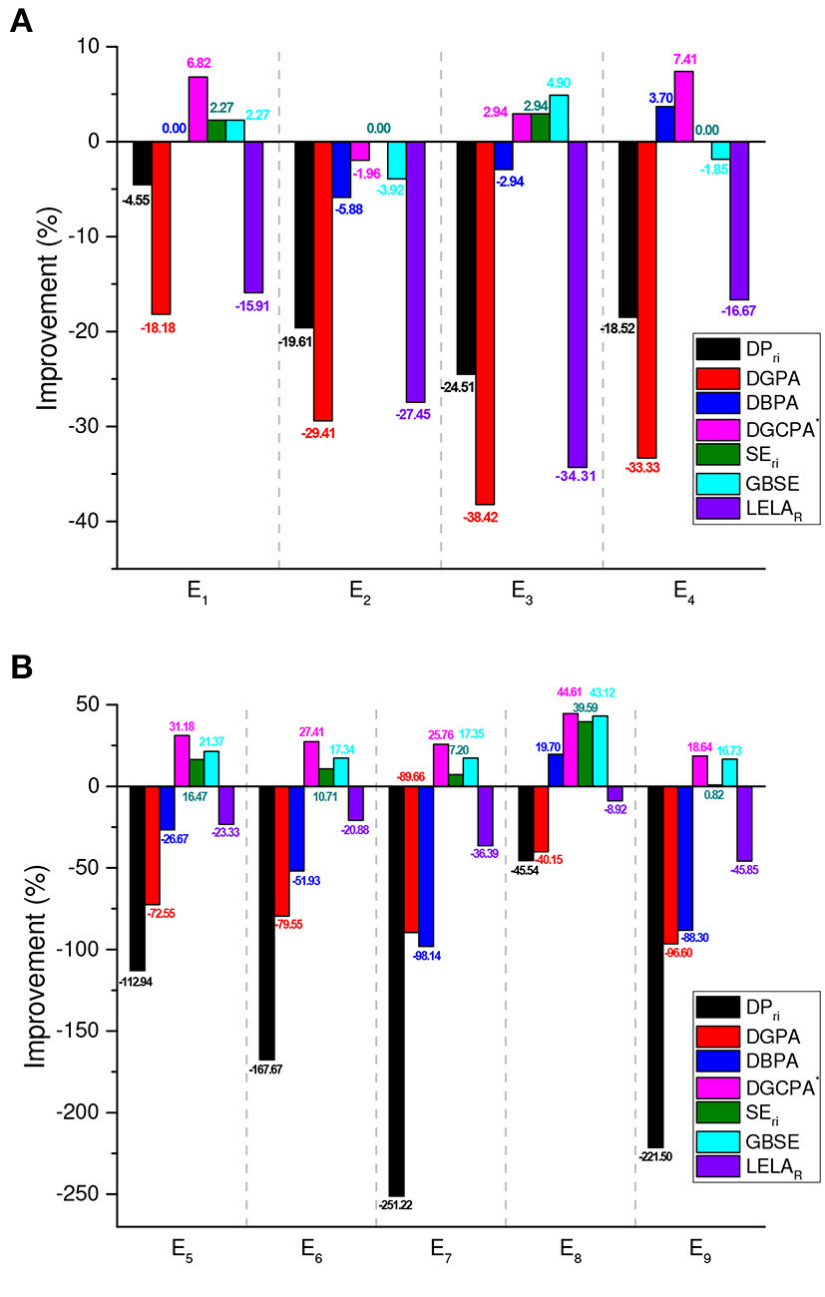
Convergence rate improvements of the compared algorithms relative to PFLA in benchmark environments, calculated by using $\frac{{\text{Iterations}}_{\text{\{PFLA\}}}-{\text{Iterations}}_{\text{\{ComparedAlgorithm\}}}}{{\text{Iterations}}_{\text{\{PFLA\}}}}$. **(A)** Two-action environments. **(B)** Ten-action environments.

In the aspect of convergence rate, obviously, in Table 3, PFLA is outperformed by the top performers, namely SE_*ri*_, GBSE, and DGCPA^*^. Figure 3 depicts the improvements of the competitors over PFLA. Take *E*_7_ as an example, the convergence rate of PFLA is improved by DGCPA^*^, SE_*ri*_, and GBSE with 25.76, 7.20, and 17.35%, respectively. While other schemes, DP_*ri*_, DGPA, and LELA_*R*_ are outperformed by PFLA significantly. Generally speaking, FPLA is faster than deterministic estimator-based schemes and slower than stochastic estimator-based algorithms.

However, taking the parameter tuning cost of the competitors into consideration, the parameter-free property begins to show its superiority. In order to clarify that point, we record the number of interactions between automaton and environment during the process of parameter tuning for each parameter-based scheme. The results are summarized in Table 4^5^. It can be seen that the extra interactions required for parameter tuning by deterministic estimator-based schemes (DGPA, DBPA, and LELA_*R*_, except DP_*ri*_) are slightly less than stochastic estimator-based schemes (DGCPA^*^, SE_*ri*_, and GBSE). Both families of schemes cost millions of extra interactions for seeking the *best parameter*. The proposed scheme can achieve a comparative performance without relying on any extra interactions/information.

**Table 4:** The parameter tuning cost (number of extra interactions) of the compared algorithms in environments *E*_1_–*E*_9_.

**Env**.	**DP_*ri*_**	**DGPA**	**DBPA**	**DGCPA**	**SE_*ri*_**	**GBSE**	**LELA_*R*_**
*E*_1_	3.075 × 10^6^	3.023 × 10^6^	2.523 × 10^6^	3.046 × 10^7^	2.062 × 10^7^	1.881 × 10^7^	2.471 × 10^6^
*E*_2_	3.866 × 10^6^	4.552 × 10^6^	3.373 × 10^6^	3.633 × 10^7^	2.669 × 10^7^	2.241 × 10^7^	3.346 × 10^6^
*E*_3_	1.521 × 10^7^	1.554 × 10^7^	1.045 × 10^7^	9.192 × 10^7^	8.704 × 10^7^	6.180 × 10^7^	1.042 × 10^7^
*E*_4_	2.616 × 10^6^	5.331 × 10^6^	2.147 × 10^6^	3.445 × 10^7^	2.215 × 10^7^	2.025 × 10^7^	2.362 × 10^6^
*E*_5_	3.947 × 10^8^	6.248 × 10^7^	1.033 × 10^8^	2.421 × 10^8^	1.268 × 10^9^	3.443 × 10^8^	2.437 × 10^7^
*E*_6_	1.813 × 10^9^	1.708 × 10^8^	4.117 × 10^8^	7.442 × 10^8^	6.905 × 10^9^	9.331 × 10^8^	6.262 × 10^7^
*E*_7_	1.503 × 10^10^	1.369 × 10^9^	4.931 × 10^9^	7.618 × 10^9^	1.207 × 10^11^	9.158 × 10^9^	3.910 × 10^8^
*E*_8_	2.008 × 10^8^	5.264 × 10^7^	4.146 × 10^7^	1.808 × 10^8^	7.079 × 10^8^	2.714 × 10^8^	2.209 × 10^7^
*E*_9_	1.802 × 10^9^	1.208 × 10^8^	5.933 × 10^8^	5.495 × 10^8^	6.266 × 10^9^	8.092 × 10^8^	7.029 × 10^7^

**Figure 4:**
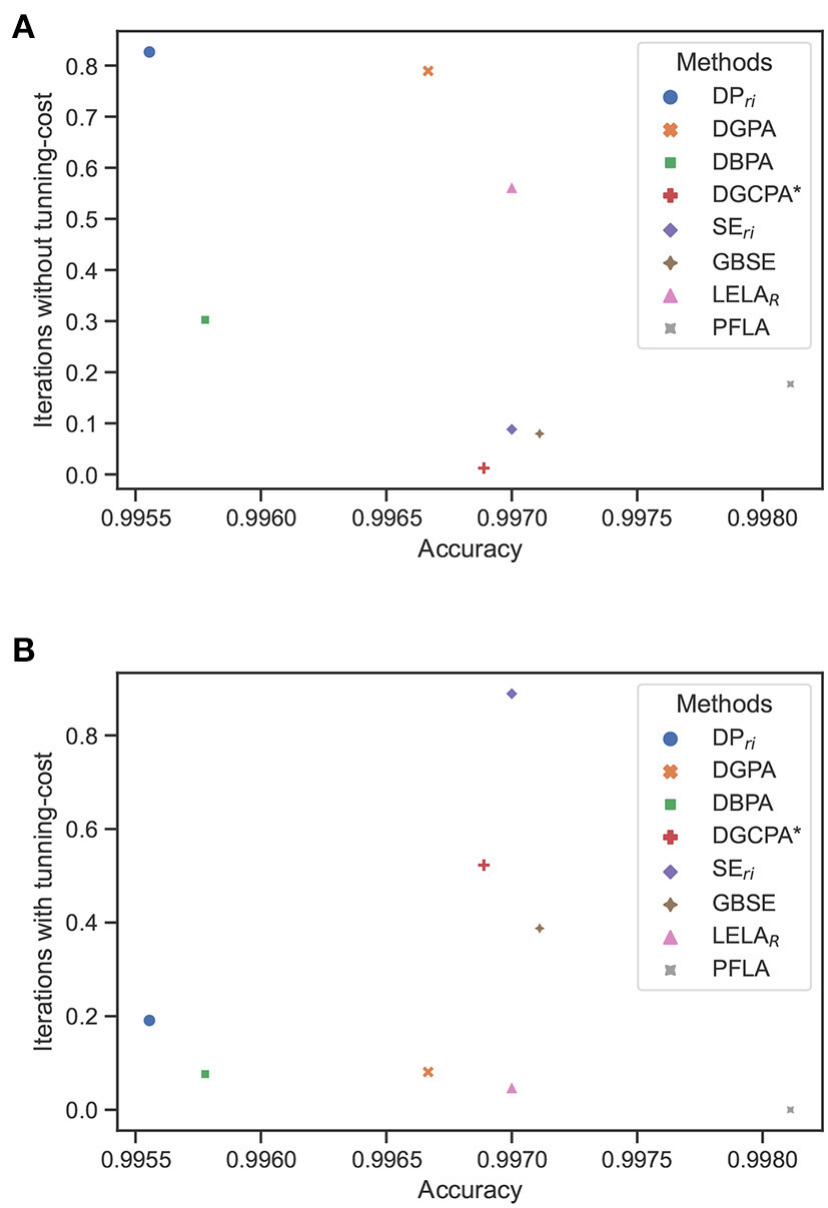
Averaged iterations vs. averaged accuracy in the nine benchmark environments of compared methods. **(A)** Without considering the parameter tunning cost. **(B)** With consideration of the parameter tunning cost.

For better visualization, a scatter map is used to illustrate the performance of different methods. In the scatter map, each dot represents a specific method discussed in this section. The x-axis indicates the averaged accuracy achieved by each method in the benchmark environments, and the y-axis indicates the averaged iterations need for each method to get converged in the benchmark environments, as shown in Figure 4. It is noted that the iterations are normalized with respect to each environment before being averaged over different environments for each method. As we are always pursuing a method with higher accuracy and convergence rate, the method approaching the right bottom corner of the figure is better than the others. From Figure 4B, we can draw the conclusion that taking the parameter tuning cost of the competitors into consideration, the proposed PFLA is the best of all competitors.

### 5.2. Comparison with untuned schemes

In this part, the parameter-based algorithms are simulated in benchmark environments without their learning parameter specifically tuned. Their performance will be compared with PFLA under the same condition—no extra information about the environment is available.

#### 5.2.1. Using generalized learning parameter

Firstly, the best parameter in *E*_2_ and *E*_6_ are applied for learning in other environments respectively to evaluate how well they can “generalize” in other environments. The results are shown in Tables 5, 6, respectively.

**Table 5:** Comparison of convergence rate and accuracy of the parameter-based algorithms in all environments other than *E*_2_, when using the “best” learning parameters in *E*_2_.

**Env**.	**DP**_*****ri*****_		**DGPA**		**DBPA**		**DGCPA**		**SE**_*****ri*****_		**GBSE**		**LELA**_*****R*****_	
	**Iter**.	**Acc**.	**Iter**.	**Acc**.	**Iter**.	**Acc**.	**Iter**.	**Acc**.	**Iter**.	**Acc**.	**Iter**.	**Acc**.	**Iter**.	**Acc**.
*E*_1_	55	0.998	65	0.999	49	0.998	53	0.995	47	0.999	48	0.999	59	0.998
*E*_3_	63	0.975	70	0.976	57	0.976	61	0.972	57	0.979	62	0.983	67	0.976
*E*_4_	90	0.999	66	0.996	79	0.999	45	0.994	69	0.999	80	0.999	96	0.999
*E*_5_	264	0.895	767	0.995	301	0.967	640	0.999	286	0.962	701	0.998	1,026	0.999
*E*_6_	319	0.804	835	0.971	408	0.927	821	0.996	351	0.897	836	0.987	1,068	0.995
*E*_7_	393	0.658	976	0.858	577	0.806	1,249	0.957	443	0.748	1,093	0.905	1,155	0.909
*E*_8_	253	0.918	752	0.997	280	0.979	616	0.999	273	0.981	648	0.999	954	0.999
*E*_9_	246	0.801	827	0.981	275	0.869	751	0.997	299	0.903	636	0.984	758	0.986

**Table 6:** Comparison of convergence rate and accuracy of the parameter-based algorithms in all environments other than *E*_6_, when using the “best” learning parameters in *E*_6_.

**Env**.	**DP**_*****ri*****_		**DGPA**		**DBPA**		**DGCPA**		**SE**_*****ri*****_		**GBSE**		**LELA**_*****R*****_	
	**Iter**.	**Acc.^*a*^**	**Iter**.	**Acc**.	**Iter**.	**Acc**.	**Iter**.	**Acc**.	**Iter**.	**Acc**.	**Iter**.	**Acc**.	**Iter**.	**Acc**.
*E*_1_	812	1	125	0.999	282	1	29	0.783	95	1	26	0.769	61	0.999
*E*_2_	923	1	126	0.999	320	1	28	0.777	103	0.999	29	0.781	68	0.998
*E*_3_	899	1	133	0.996	317	1	31	0.725	124	0.998	29	0.716	70	0.978
*E*_4_	1572	1	126	0.999	535	1	28	0.791	137	1	46	0.846	101	0.999
*E*_5_	1879	0.999	1,582	0.999	969	0.999	501	0.972	641	0.999	599	0.999	1,085	0.999
*E*_7_	3961	0.942	1,939	0.945	2,358	0.965	1,055	0.929	1,203	0.942	1,110	0.948	1,219	0.917
*E*_8_	1641	0.999	1,555	0.999	845	0.999	495	0.974	629	0.999	592	0.999	1008	0.999
*E*_9_	1923	0.987	1,667	0.998	1,078	0.988	673	0.973	725	0.996	675	0.997	799	0.989

a Acc., Accuracy.

#### 5.2.2. Using random learning parameter

Secondly, randomly selected learning parameters are adopted to evaluate the expected performance of each algorithm in fully unknown environments. The random resolution parameter takes value in the range from 90% of the minimal value to 110% of the maximal value of the best resolution parameter in the nine benchmark environments^6^, and a range from 1 to 20 for the perturbation parameter if needed. The simulation results are demonstrated in Table 7.

**Table 7:** Comparison of the average number of iterations required for convergence of the parameter-based algorithms in environments *E*_1_ to *E*_9_.

**Env**.	**DP**_*****ri*****_		**DGPA**		**DBPA**		**DGCPA**		**SE**_*****ri*****_		**GBSE**		**LELA**_*****R*****_	
	**Iter**.	**Acc**.	**Iter**.	**Acc**.	**Iter**.	**Acc**.	**Iter**.	**Acc**.	**Iter**.	**Acc**.	**Iter**.	**Acc**.	**Iter**.	**Acc**.
*E*_1_	1,606	0.999	216	0.999	574	0.999	76	0.924	121	0.999	71	0.943	108	0.999
*E*_2_	1,824	0.999	217	0.999	652	0.999	73	0.922	132	0.999	78	0.943	121	0.999
*E*_3_	1,767	0.999	224	0.996	638	0.998	87	0.896	152	0.996	95	0.927	124	0.990
*E*_4_	3,121	0.999	217	0.999	1,105	0.999	66	0.928	189	0.999	109	0.953	192	0.999
*E*_5_	3,253	0.996	2,821	0.999	1,476	0.997	836	0.977	687	0.993	925	0.997	2,153	0.999
*E*_6_	3,995	0.988	2,922	0.995	2,072	0.993	1,042	0.975	886	0.979	1,158	0.991	2,229	0.996
*E*_7_	6,260	0.951	3,309	0.963	3,626	0.970	1,647	0.952	1,302	0.911	1,699	0.952	2,395	0.958
*E*_8_	2,859	0.997	2,774	0.999	1,288	0.999	810	0.978	647	0.996	878	0.998	2003	0.999
*E*_9_	3,031	0.985	2,919	0.997	1,615	0.987	1,041	0.976	768	0.979	988	0.988	1547	0.993

The randomly selected learning parameters are used and 250,000 experiments were performed for each scheme in each environment.

From the three tables, there is a significant decline in accuracy in some environments. As the accuracies differ greatly in those cases, the convergence rates cannot be compared directly. Still, several conclusions can be drawn. One is that the performance of untuned parameter-based algorithms is unstable when learning in an unknown environment, and thus cannot be used in practical applications without parameter tuning. Another conclusion is that those algorithms, that use generalized learning parameters or random learning parameters, are either have a lower accuracy or a slower convergence rate than PFLA in the benchmark environment. In other words, none of them can outperform PFLA in both accuracy and convergence rate without the help of prior information.

### 5.3. Discussion of the fairness of the comparison

Technically speaking, the comparison between PFLA and well-tuned schemes is not fair. This is because the interactions can be perceived as information exchanges between automaton and the environment. So if the number of interactions is unlimited, the algorithm can simply use the empirical distributions. The outperforming of the well-tuned schemes owes to their richer knowledge about the environment acquired during the parameter tuning process. And for this reason, a fair comparison between PFLA and untuned schemes is carried out. Despite the unfairness of the first comparison, the significance lies in providing baselines for evaluating the convergence rate of PFLA qualitatively.

By the way, the comparison within parameter-based algorithms is not fair either, because the amount of prior information acquired is different. This method is widely used by the research community to compare the theoretically best performance of their proposed algorithms, however, the hardness of the algorithm can achieve theoretically best is usually ignored.

## 6. Conclusion

In this paper, we propose a parameter-free learning automaton scheme for learning in stationary stochastic environments. The proof of the ε-optimality of the proposed scheme in every stationary random environment is presented. Compared with existing schemes, the proposed PFLA possesses a parameter-free property, i.e., a set of parameters that can be universally applicable to all environments. Furthermore, our scheme is evaluated in four two-action and five 10-action benchmark environments and compared with several classic and state-of-the-art schemes in the field of LA. Simulations confirm that our scheme can converge to the optimal action with high accuracy. Although the rate of convergence is outperformed by some schemes that are well-tuned for specific environments, the proposed scheme still shows its intriguing property of not relying on the parameter-tuning process. Our future work includes optimizing the exploration strategy further.

## Data availability statement

The original contributions presented in the study are included in the article/supplementary material, further inquiries can be directed to the corresponding author.

## Author contributions

All authors listed have made a substantial, direct, and intellectual contribution to the work and approved it for publication.

## Funding

This research work was funded by the National Nature Science Foundation of China under Grant 61971283, Shanghai Municipal Science and Technology Major Project under Grant 2021SHZDZX0102, and Shanghai AI Innovation and Development Project under Grant 2020-RGZN-02026.

## Conflict of interest

Author HG was employed by company Shanghai Data Miracle Intelligent Technology Co., Ltd. The remaining authors declare that the research was conducted in the absence of any commercial or financial relationships that could be construed as a potential conflict of interest.

## Publisher's note

All claims expressed in this article are solely those of the authors and do not necessarily represent those of their affiliated organizations, or those of the publisher, the editors and the reviewers. Any product that may be evaluated in this article, or claim that may be made by its manufacturer, is not guaranteed or endorsed by the publisher.

## Footnotes

^1^For this reason, the terms *convergence rate* and *iteration* are used interchangeably.

^2^*E*_1_ defined in Papadimitriou et al. (2004) corresponds to *E*_5_ defined in Section 5 of this paper.

^3^The details will be elaborated in Section 5.

^4^Technically speaking, the comparison is not completely fair, that's the reason the word “fairly” are quoted. An explanation will be given in later subsections.

^5^It is noted that the numerical value shown in Table 4 may differ according to the way parameter tuning being implemented, still it gives qualitatively evidence to the heavy parameter tuning cost of the parameter-based schemes. The technical details of the parameter tuning procedure used here are provided in the Appendix.

^6^For example, the resolution parameter of DPri is range from ⌊90% ∗ 18⌋ to ⌈110% ∗ 2356⌉, i.e., from 16 to 2,592.

## Appendix

### The standard parameter tuning procedure of learning automata

As emphasized in Section 1, parameter tuning is intended to balance the trade-off between speed and accuracy. And the standard procedure of parameter tuning is pioneered in Oommen and Lanctôt (1990) and become a common practice in follow-up researches (Oommen and Agache, 2001; Agache and Oommen, 2002; Papadimitriou et al., 2004; Zhang et al., 2014; Ge et al., 2015a; Jiang et al., 2015).

The basic idea is, that the smallest value of resolution parameter *n* that yielded the fastest convergence and that simultaneously resulted in zeros errors in a sequence of *NE* experiments are defined as “best” parameters. Besides, to reduce the variance coefficient of the “best” values of *n*, Papadimitriou et al. (2004) advocate performing the same procedure 20 times and computing the average “best” value of *n* in these experiments. For tuning stochastic estimator-based learning automata, which have a perturbation parameter γ in addition to the well-known resolution parameter *n*. A two-dimensional grid search should be performed to seek the best parameter pair (*n*, γ). The method used in Papadimitriou et al. (2004) is to obtain the “best” resolution parameter *n* for each value of γ, and then evaluate the speed of convergence for each of the (*n*, γ) pairs and choose the best pair.

Based on these instructions, we use the following procedure for parameter tuning in our experiment:

The resolution parameter is initialized to 1 and increased by 1 each time a wrong convergence emerges until a certain number of successive *No Error* experiments is carried out. Repeat this process 20 times, averaging over these 20 resulting values, and denote it as the best resolution parameter. The value of number of successive No Error experiments is set as *NE* = 750, as the same value in Papadimitriou et al. (2004), Zhang et al. (2014), Jiang et al. (2015), and Ge et al. (2015a). For tuning the “best” γ, in our simulation settings, for the four two-action environments, the search range of γ is from 1 to 10; For the five ten-action environments except *E*_7_, the search range of γ is from 1 to 20, while for *E*_7_, the most difficult one, the range is a little wider, from 1 to 30.

It is noted that the above standard procedure has been widely adopted by the research community, but it does not mean this is the most efficient way. Apparently, it can be improved by several methods, such as random search or two-stage coarse-to-fine search. This issue is worth further investigation and is beyond the scope of this paper.
